# 5-HT_7_ Receptor Is Involved in Electroacupuncture Inhibition of Chronic Pain in the Spinal Cord

**DOI:** 10.3389/fnins.2021.733779

**Published:** 2021-09-17

**Authors:** Xiao-Cui Yuan, Xiang-Ji Yan, Li-Xia Tian, Yi-Xiao Guo, Yu-Long Zhao, Sani Sa’idu Baba, Yu-Ying Wang, Ling-Li Liang, Hong Jia, Lin-Ping Xu, Li Li, Han Lin, Fu-Quan Huo

**Affiliations:** ^1^Department of Physiology and Pathophysiology, School of Basic Medical Sciences, Institute of Neuroscience, Translational Medicine Institute, Xi’an Jiaotong University Health Science Center, Xi’an, China; ^2^Key Laboratory of Environment and Genes Related to Diseases, Ministry of Education, Xi’an Jiaotong University, Xi’an, China; ^3^Key Laboratory of Anesthesiology of Zhejiang Province, Department of Anesthesiology, The Second Affiliated Hospital, Wenzhou Medical University, Wenzhou, China

**Keywords:** knee osteoarthritis (KOA), 5-HT_7_ receptor, GABA_A_ receptor, chronic pain, electroacupuncture analgesia (EAA)

## Abstract

Knee osteoarthritis (KOA) is a common and disabling condition characterized by attacks of pain around the joints, and it is a typical disease that develops chronic pain. Previous studies have proved that 5-HT_1_, 5-HT_2_, and 5-HT_3_ receptors in the spinal cord are involved in electroacupuncture (EA) analgesia. The 5-HT_7_ receptor plays antinociceptive role in the spinal cord. However, it is unclear whether the 5-HT_7_ receptor is involved in EA analgesia. The 5-HT_7_ receptor is a stimulatory G-protein (Gs)-coupled receptor that activates adenylyl cyclase (AC) to stimulate cyclic adenosine monophosphate (cAMP) formation, which in turn activates protein kinase A (PKA). In the present study, we found that EA significantly increased the tactile threshold and the expression of the 5-HT_7_ receptor in the dorsal spinal cord. Intrathecal injection of 5-HT_7_ receptor agonist AS-19 mimicked the analgesic effect of EA, while a selective 5-HT_7_ receptor antagonist reversed this effect. Moreover, intrathecal injection of AC and PKA antagonists prior to EA intervention prevented its anti-allodynic effect. In addition, GABA_A_ receptor antagonist bicuculline administered (intrathecal, i.t.) prior to EA intervention blocked the EA effect on pain hypersensitivity. Our data suggest that the spinal 5-HT_7_ receptor activates GABAergic neurons through the Gs–cAMP–PKA pathway and participates in EA-mediated inhibition of chronic pain in a mouse model of KOA.

## Introduction

Knee osteoarthritis (KOA) is a common degenerative joint disorder, which affects the quality of life of patients. The symptoms of KOA include pain, joint swelling, and limitation in range of motion (Wu et al., 2020). Our previous studies had proved that electroacupuncture (EA) is effective in relieving chronic pain in patients with KOA and animal models of KOA (Yuan et al., 2018a, b; Lv et al., 2019). The serotonergic (5-HT) system has been considered as one of the main neurotransmitter systems that plays a key role in pain transmission, processing, and modulation (Brenchat et al., 2010). Accumulating evidence indicates that 5-HT mediates the analgesic effects of EA in the spinal cord *via* 5-HT_1_, 5-HT_2_, and 5-HT_3_ receptors, which have been shown to be involved in EA-mediated analgesia (Li et al., 2011; Seo et al., 2016). However, it is still unclear whether the 5-HT_7_ receptor is involved in EA analgesia.

The 5-HT_7_ receptor had received much attention among the most recently described members of the 5-HT receptor family as reviewed by Cortes-Altamirano et al. (2018). It is a stimulatory G-protein (Gs)-coupled receptor that activates adenylyl cyclase (AC), which in turn increases the intracellular concentration of cyclic adenosine monophosphate (cAMP) and activates protein kinase A (PKA) (Cortes-Altamirano et al., 2018). A previous study has found a significant increase in the 5-HT_7_ receptor level in the spinal dorsal horn in the neuropathic pain model (Brenchat et al., 2010). Moreover, administration of 5-HT_7_ receptor agonist exerts antinociceptive effects in both neuropathic and inflammatory pain models (Brenchat et al., 2010; Yang et al., 2014). These results suggest that the 5-HT_7_ receptor plays an antinociceptive role in the spinal cord. A previous study has reported co-localization of 5-HT_7_ receptor with GABAergic neurons in the spinal cord dorsal horn, and intrathecal injection of GABA_A_ receptor antagonist bicuculline into the spinal dorsal horn completely abolished the anti-hyperalgesic effects of 5-HT_7_ receptor agonist in the chronic constriction injury to the sciatic nerve (CCI-SN) model (Viguier et al., 2012; Lin et al., 2015). It is therefore reasonable to suggest that spinal GABAergic interneurons are involved in 5-HT_7_ receptor-mediated antinociception.

In the present study, we investigated whether EA activates GABAergic neurons through the 5-HT_7_–Gs–cAMP–PKA pathway, thereby inhibiting chronic pain in a mouse model of KOA. We first determined the involvement of the 5-HT_7_ receptor in the analgesic effect of EA and then the roles of the Gs–cAMP–PKA pathway, and GABA_A_ receptor accompanying the chronic pain inhibitory effect of EA was also elucidated in the KOA model of chronic pain in male mice.

## Materials and Methods

### Ethics Statement

The animal study was approved by the Institutional Animal Care Committee of Xi’an Jiaotong University.

### Experimental Animals

Eight-week-old male C57BL/6J mice were obtained from the Medical Experimental Animal Center of Xi’an Jiaotong University, Shaanxi Province, China. The experimental protocol was in accordance with the ethical guidelines of the International Association for the Study of Pain (Zimmermann, 1983). The mice were housed in a room with controlled ambient temperature (22 ± 1°C) and under a 12-h light–dark cycle (lights on at 8 a.m.) with access to food and water *ad libitum*. All efforts were made to minimize the number of animals used, and less distress to the animals was also ensured. Mice were randomly divided into different groups.

### Induction of KOA

Knee osteoarthritis was induced by intra-articular injection of monosodium iodoacetate (MIA; Sigma, United Kingdom) into the left knee joint after mice were briefly anesthetized with isoflurane. The knee joint was shaved and flexed at a 90° angle. Five microliters of 5 mg/ml MIA in sterile saline (0.9%) were injected into the joint space of the left knee through the infrapatellar ligament with a 30-gauge needle (La Porta et al., 2013). The choice of MIA concentration was made based on its adequacy to elicit histological changes in the cartilage (Yuan et al., 2018a) and induce joint pain (Harvey and Dickenson, 2009) in mice. Control group mice were made to receive an intra-articular injection of vehicle (5 μl of sterile saline, 0.9%) as placebo.

### Electroacupuncture Treatment

The animals were habituated to the restriction bag for 30 min each day for 3 days before KOA induction. In the EA treatment group, mice received EA administration on the left “Neixiyan” (Ex-LE4) and “Dubi” (ST35) once every other day for 4 weeks, starting from 2 or 18 days after MIA injection. EA with operation specifications of 1 mA and 0.1 ms at 2 Hz for 30 min was administered (Yuan et al., 2018b), and the current was delivered with a Han’s Acupoint Nerve Stimulator (LH202; Huawei Co., Ltd., Beijing, China). Two acupuncture needles were inserted into two acupoints corresponding to Ex-LE4 and ST35 in humans. Ex-LE4 is located at the medial cavity of the patella and the patellar ligament, and ST35 lies on the lateral cavity of the patella and patellar ligament. Ex-LE4 and ST35 were chosen, because they are specific acupoints for treating knee problems (Ng et al., 2003; Selfe and Taylor, 2008).

For Sham treatment control, acupuncture needles were inserted bilaterally into Ex-LE4 and ST35 without electrical stimulation and manual needle manipulation, such as lifting, inserting, twisting, etc. This procedure is comparable to actual treatment but produces little therapeutic effect. Our previous study showed that needles inserted into active acupoints, but given no electrical or manual stimulation, do not produce analgesia (Yuan et al., 2018b).

### Mechanical Paw Withdrawal Threshold Measurement

Mechanical allodynia was also demonstrated in the hind paw of animals with KOA, using von Frey filaments (Fernihough et al., 2004; La Porta et al., 2013). The behavioral test was performed three times before KOA induction and once every other day, starting from the first day to 4 weeks after KOA induction. The animals were habituated to the testing environment for 30 min.

Mechanical allodynia was assessed by placing mice on an elevated mesh floor, and the tactile threshold was measured by using the “up-down” method (Chaplan et al., 1994). After an acclimation period of 30 min, a series of calibrated von Frey filaments (Stoelting, Kiel, WI, United States) was applied perpendicularly to the plantar surface of the left hind paw with sufficient force to bend the filament for 6 s. Brief withdrawal or paw flinching was considered as a positive response. The test was repeated two times in mice, and the mean value of each mouse was calculated and recorded.

### Quantitative Polymerase Chain Reaction

The dorsal spinal cord tissues were removed 4 weeks after vehicle or MIA injection. The tissues were excised from mice immediately after the animals were anesthetized with 10% chloralic hydras and decapitated. Total RNA was isolated from the spinal cord tissues by using RNAeasy^TM^ Animal RNA Isolation Kit with Spin Column (Beyotime Biotechnology, Nanjing, China). Aliquots of 2 μg total RNA were reverse transcribed into cDNA using ReverTraAce-a-^TM^ (Toyobo, Japan). The PCR mixture (10 μl) consisted of 1 μl diluted cDNA, 5 μl SYBR quantitative polymerase chain reaction (qPCR) mix (Takara, Japan), 1 μl of each primer, and 3 μl of water. CFX96 system (Bio-Rad, United Kingdom) was used to perform qPCR, and the relative expression levels were quantified with CFX Manager software. The expression level of each gene was determined by the threshold cycle (CT). The amount of target mRNAs, normalized to the endogenous control (GAPDH), was obtained by 2^–ΔΔCT^. Results of independent experiments were expressed as the percentage change over mRNA level of the control group. Specific sequence primers are listed as follows: mouse-*5-HT_7_* forward: 5′-GGCCTGAGAGAAGCGAGTTT-3′; reverse: 5′-TCACTCCGTTACCCCAAGGT-3′. Mouse-*GAPDH* forward: 5′-AGGTCGGTGTGAACGGATTTG-3′; reverse: 5′-TGTAGACCATGTAGTTGAGGTCA-3′.

### Western Blotting

The dorsal spinal cord tissues were removed as described above. The tissues were also homogenized in 200 μl RIPA lysis buffer (Beyotime Biotechnology, China) and 2 mM phenylmethylsulfonyl fluoride and then centrifuged at 12,000 × *g* for 15 min. The pellet was discarded, and protein concentrations of the supernatant were determined by using the BCA Protein Assay Kit (Pioneer Biotechnology, China). Proteins were denatured with sodium dodecyl sulfate-polyacrylamide gel electrophoresis (SDS-PAGE) loading buffer at 95°C for 5 min and separated on glycine–SDS-PAGE gel. The proteins were transferred onto a polyvinylidene fluoride membrane and blocked for 1 h by 5% non-fat dry milk in Tris-buffered saline (TBS) containing 0.1% Tween-20. The membrane was incubated with rabbit anti-5-HT_7_ antibody (1:1,000, Novus Biologicals, United States) or mouse anti-β-actin antibody (1:5,000; Proteintech, United States) at 4°C overnight. After they have been washed three times in 0.1% TBS–Tween 20 (pH 7.6), the membranes were then incubated with horseradish peroxidase-conjugated secondary antibodies from Merck Millipore: goat anti-rabbit secondary antibody (1:5,000) or goat anti-mouse secondary antibody (1:5,000) for 2 h at room temperature and also washed three times. The enhanced chemiluminescence method (ECL Plus Western Blotting detection reagents, Merck Millipore, United States) was used to reveal the protein bands captured by the ChampChemi System with SageCapture software (Sagecreation Service for Life Science, Beijing, China). The band intensity was quantified and analyzed by Image J software. β-Actin was used as the internal control. The values of 5-HT_7_ were expressed as the ratio of the optical density of band to the density of the related β-actin band. The values were further normalized *via* dividing the average value each of the control group.

### Drug Dosing and Administration

Drugs were administered [intrathecal (i.t.), 10 μl] for 30 min before EA treatment. Intrathecal delivery of drugs were performed under isoflurane anesthesia by lumbar puncture. The needle was briefly inserted into the L5 to L6 intervertebral space through the vertebral column. A successful needle insertion was determined by a tail flick response (Hylden and Wilcox, 1980). Each drug was slowly injected over a 10-s period. Then, the needle was carefully removed from the spinal cord. The drug control groups underwent the same procedure to receive an identical injection of vehicle without drug.

Drugs used in this study were purchased from Tocris Cookson (Bristol, United Kingdom) or RBI/Sigma (St. Louis, MO, United States) and include the 5-HT_7_ receptor agonist AS-19 [(2S)-(+)-5-(1,3,5-trimethylpyrazol-4-yl)-2-(dimethylamino) tetralin, 20 μg], 5-HT_7_ receptor antagonist SB-269970 [(R)-3-(2-(2-(4-methyl-piperidin-1-yl)ethyl)-pyrrolidine-1-sulphonyl)-phenol, 10 μg], AC inhibitor SQ22536 [9-(tetrahydro-2-furanyl)-9H-purin-6-amine, 2.8 μg], and PKA inhibitor H89 {N-[2-(p-bromocinnamylamino) ethyl]-5-isoquinolinesulfonamide dihydrochloride, 2.5 μg}. The drugs were freshly prepared in saline, 1% dimethyl sulfoxide (DMSO), or 10% DMSO. The doses of drugs were chosen based on their proven effectiveness at that particular doses by other studies (Dogrul et al., 2012; Li et al., 2015; Yesilyurt et al., 2015; Zhou et al., 2015; Feng et al., 2017) and our preliminary experiment. Our previous study has found that saline or 10% DMSO injected into the spinal cord did not influence the nociceptive response of rats. Equal volumes of 1% DMSO or 10% DMSO were injected into the spinal cord as vehicle controls in the present study.

### Statistical Analysis

GraphPad Prism 5.0 was used to conduct statistical analyses in this study. To determine the statistical difference in the withdrawal thresholds, we used two-way repeated-measure ANOVA where each factor has a “between subjects” (group) or “repeated measures” factor (time points), followed by a Bonferroni *post hoc* analysis of multiple comparisons. Other data were analyzed by one-way ANOVA followed by Newman–Keuls *post hoc* test with two-tailed hypothesis. Data were presented as means ± SEM. A *P*-value less than 0.05 was considered significant.

## Results

### EA Effectively Reduces Pain Hypersensitivity in KOA Mice

**FIGURE 1:**
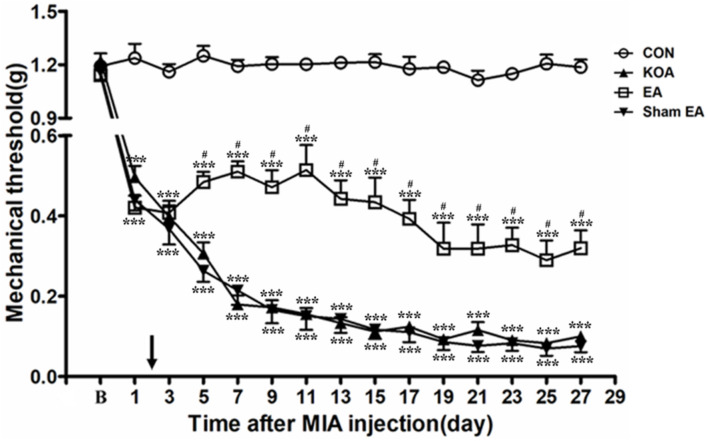
Time course of the effect of electroacupuncture (EA) on pain hypersensitivity in knee osteoarthritis (KOA) mice. Time course of tactile threshold in response to von Frey filaments in control (CON), KOA, EA, and Sham EA mice. EA was administered for 30 min, once every other day for 4 weeks, starting from 2 days after monosodium iodoacetate (MIA) injection, as indicated by a black arrow. Data are expressed as means ± SME (*n* = 8 mice in each group). ***P < 0.001, compared with the CON group; ^#^*P* < 0.05, compared with the KOA group.

Four groups of mice [control (CON), KOA, EA, and Sham EA] were used for this series of experiments. There was no significant difference in the baseline withdrawal threshold between different groups before MIA injection. Induction of KOA gradually reduced mechanical withdrawal threshold, and EA significantly increased the tactile threshold after KOA modeling (*P* < 0.001; Figure 1), consistent with our previous results (Yuan et al., 2018a, b).

As shown in Figure 1, the time course curves (i.e., CON, KOA, EA, and Sham EA-treated groups) were significantly different between treatments [*F*_(3,420)_ = 2,192.01; *P* < 0.0001], across times [*F*_(14,420)_ = 107.18; *P* < 0.0001], and for their interactions [*F*_(42,420)_ = 14.20; *P* < 0.0001]. Further analyses showed that the mechanical thresholds in the KOA group were significantly less than those in the EA-treated group from the 5th to 27th day after MIA injection (*P* < 0.05; Figure 1). However, no significant difference was observed between the Sham EA-treated group and the KOA group after MIA injection (*P* > 0.05; Figure 1).

### EA Reverses the Reduction of 5-HT_7_ Receptor Expression in the Dorsal Spinal Cord of KOA Mice

**FIGURE 2:**
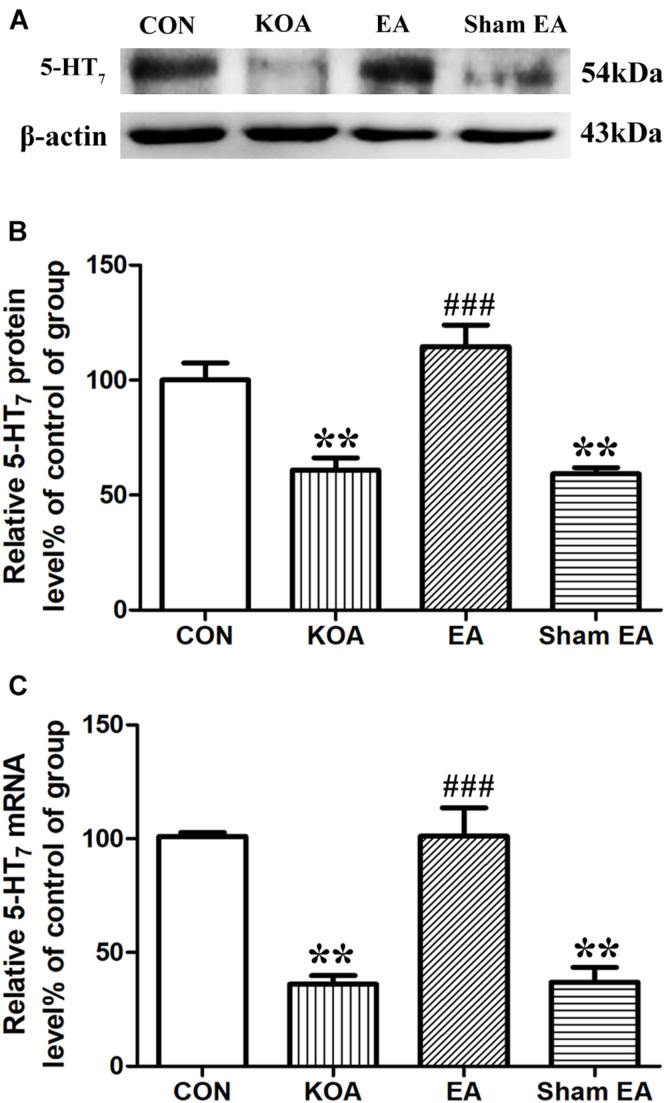
Quantitative analysis of protein and mRNA levels of 5-HT_7_ receptor in the dorsal spinal cord tissues. **(A)** Representative gel image shows the protein level of 5-HT_7_ receptor in dorsal spinal cord tissues obtained from control (CON), KOA, KOA treated with EA, and KOA treated with Sham EA. β-Actin was used as a loading control. The protein band at 54 kDa corresponds to the 5-HT_7_ receptor. **(B)** Summary data show the effect of KOA, EA, and Sham EA on the protein level of 5-HT_7_ receptor in dorsal spinal cord tissues. **(C)** Effects of KOA, EA, and Sham EA on the mRNA level of 5-HT_7_ receptor in dorsal spinal cord tissues. Data are expressed as means ± SME (*n* = 5 mice in each group). **P < 0.01, compared with the CON group; ^###^*P* < 0.001, compared with the KOA group.

The 5-HT_7_ receptor protein bands were presented in the dorsal spinal cord tissues (Figure 2A). There was a significant reduction in 5-HT_7_ receptor protein level in the dorsal spinal cord compared to that of the control group 4 weeks after KOA induction (*P* < 0.001; Figure 2B). Conversely, there was significant increase in 5-HT_7_ receptor level in the dorsal spinal cord of the KOA group compared to that of the control group (*P* < 0.001; Figure 2B) after the EA intervention.

Similarly, the mRNA level of 5-HT_7_ receptor in the KOA group was significantly lower than that in the control group 4 weeks after KOA induction (*P* < 0.001; Figure 2C). EA significantly increased the mRNA level of 5-HT_7_ receptor compared with that in the KOA group (*P* < 0.001; Figure 2C). Sham EA had no significant effect on 5-HT_7_ receptor protein and mRNA levels of KOA mice (*P* > 0.05; Figures 2B,C).

### Intrathecal Administration of a Selective 5-HT_7_ Receptor Antagonist SB-269970 Reversed the EA Effect on Mechanical Allodynia

**FIGURE 3:**
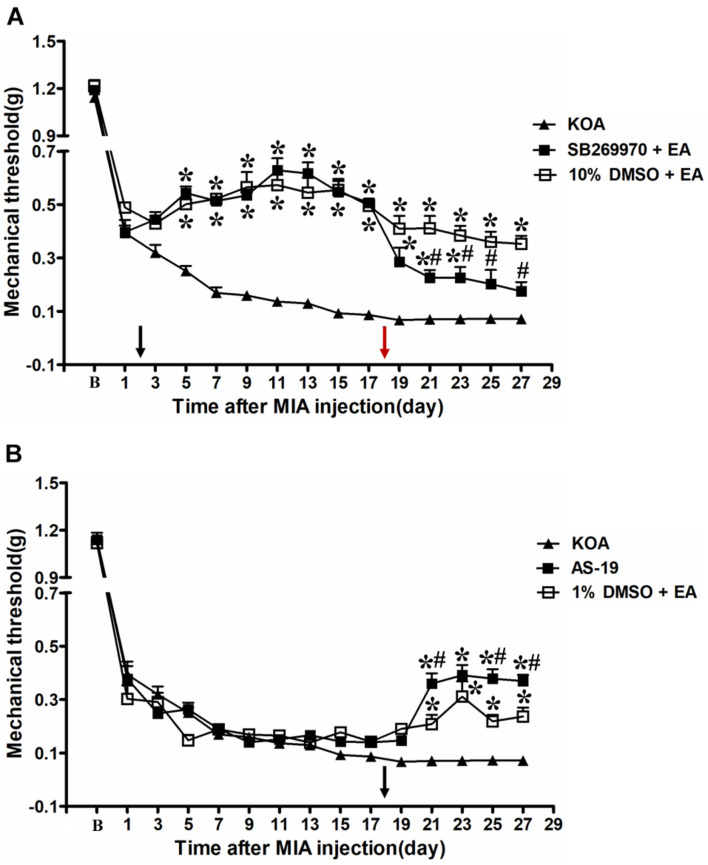
Time course of the effect of 5-HT_7_ receptor antagonist/agonist on pain hypersensitivity. **(A)** Time course of tactile threshold in response to von Frey filaments in KOA, 10% dimethyl sulfoxide (DMSO) + EA, and SB-269970 + EA mice. EA was administered for 30 min, once every other day for 4 weeks, starting from 2 days after MIA injection, as indicated by a black arrow. The 5-HT_7_ receptor antagonist SB-269970 (10 μg) was administered i.t. 30 min before EA starting from 18 days after MIA injection, once every other day for five times, as indicated by a red arrow. **(B)** Time course of the effect of EA or 5-HT_7_ receptor agonist AS-19 on tactile withdrawal threshold of KOA mice. The 5-HT_7_ receptor agonist AS-19 (20 μg) was administered i.t. or EA stimulation starting from 18 days after MIA injection, once every other day for five times, as indicated by a black arrow. Data are expressed as means ± SME (*n* = 8 mice in each group). *P < 0.05, compared with the KOA group; ^#^*P* < 0.05, compared with the 10% DMSO + EA (or 1% DMSO + EA) group.

Our previous studies have demonstrated that the chronic pain stage started from the 17th day after KOA induction (Yuan et al., 2018b). Therefore, we administered a selective 5-HT_7_ receptor antagonist SB-269970 (10 μg, i.t.) 30 min before EA treatment on the 18th day after KOA induction, once every other day for five times. It was found that 10% DMSO did not influence the effect of EA on the mechanical thresholds (Figure 3A).

However, SB-269970 (10 μg, i.t.), a selective 5-HT_7_ receptor antagonist, reversed the EA effect on the tactile withdrawal thresholds. As shown in Figure 3A, the time course curves (i.e., KOA, 10% DMSO plus EA, and SB-269970 plus EA groups) differed significantly from the treatments [*F*_(2,__315)_ = 384.03; *P* < 0.0001], across times [*F*_(14,__315)_ = 159.05; *P* < 0.0001], and for their interactions [*F*_(28,__315)_ = 8.17; *P* < 0.0001]. Further analyses showed that the mechanical thresholds in the SB-269970 plus EA-treated group were significantly less than those in the 10% DMSO plus EA group from the 21st to 27th day after KOA induction (*P* < 0.05; Figure 3A). Moreover, the mechanical thresholds in the SB-269970 plus EA-treated group were also significantly higher than those in the KOA group from the 1st to 5th day after the first SB-269970 injection (from the 19th to 23rd day after KOA induction) (*P* < 0.05; Figure 3A).

### Intrathecal Administration of a Selective 5-HT_7_ Receptor Agonist AS-19 Mimicked the EA Effect on Mechanical Allodynia

The 5-HT_7_ receptor agonist AS-19 (20 μg, i.t.) was administered from the 18th day after KOA induction. Meanwhile, EA stimulation started from the same day. Both interventions were administered once every other day for five times. The AS-19 group markedly increased the mechanical thresholds, as well as the 1% DMSO plus EA-group (*P* < 0.001).

As shown in Figure 3B, the time course curves for these groups (AS-19, KOA, and 1% DMSO plus EA treated) significantly differed from the treatment group [*F*_(2,__315)_ = 45.81; *P* < 0.0001], across times [*F*_(14,__315)_ = 269.81; *P* < 0.0001], and their interactions [*F*_(28,__315)_ = 8.12; *P* < 0.0001]. Further analyses showed that the mechanical thresholds in the AS-19-treated group were significantly higher than those in the KOA group from the 21st to 27th day after KOA induction (*P* < 0.001; Figure 3B). Also, the EA treatment significantly increased the mechanical thresholds from the 21st to 27th day after KOA induction (*P* < 0.01; Figure 3B, 1% DMSO + EA). Moreover, the mechanical thresholds in the AS-19-treated group were significantly higher than those in the 1% DMSO plus EA group on the 21st, 25th, and 27th day after KOA induction (*P* < 0.01; Figure 3B).

### Intrathecal AC Inhibitor SQ22536 Reversed the EA Effect on Mechanical Allodynia

The 5-HT_7_ receptor stimulates cAMP formation by activation of AC when coupled to Gs, which in turn activates PKA (Cortes-Altamirano et al., 2018). Unpublished data from our laboratory has revealed analgesic behavior of 5-HT_7_–Gs–cAMP–PKA pathway in neuropathic pain model. Therefore, we use AC and PKA inhibitors to explore the possible involvement of Gs–cAMP–PKA pathway in the EA inhibition of chronic pain in a mouse model of KOA.

**FIGURE 4:**
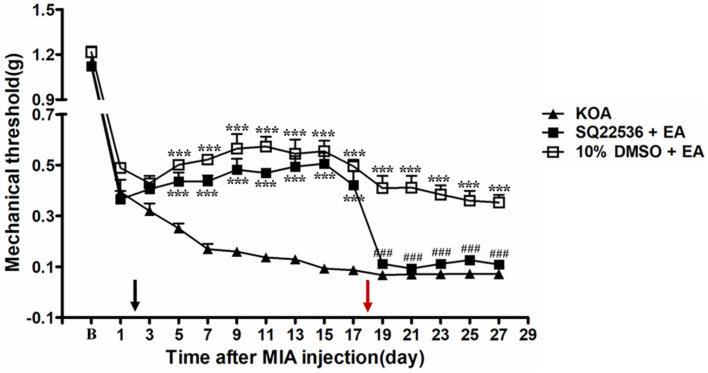
The effect of adenylyl cyclase (AC) inhibitor SQ22536 on EA analgesia. Time course of the effect of AC inhibitor SQ22536 on tactile withdrawal threshold of EA mice. EA was administered for 30 min, once every other day for 4 weeks, starting from 2 days after MIA injection, as indicated by a black arrow. The AC inhibitor SQ22536 (2.8 μg) was administered i.t. 30 min before EA starting from 18 days after MIA injection, once every other day for five times, as indicated by a red arrow. Data are expressed as means ± SME (*n* = 8 mice in each group). ***P < 0.001, compared with the KOA group; ^###^*P* < 0.001, compared with the 10% DMSO + EA group.

Adenylyl cyclase inhibitor SQ22536 (2.8 μg, i.t.) was administered 30 min before EA intervention starting from the 18th day after KOA induction, once every other day for five times. AC inhibitor SQ22536 was found to reverse the EA effect on the tactile withdrawal thresholds (*P* < 0.001). As shown in Figure 4, the time course curves for these groups (KOA, SQ22536 plus EA, and 10% DMSO plus EA treated) significantly differed from treatments [*F*_(2,__315)_ = 377.77; *P* < 0.0001], across times [*F*_(14,__315__)_ = 184.26; *P* < 0.0001], and for their interactions [*F*_(28,__315)_ = 9.18; *P* < 0.0001]. Further analyses showed that the mechanical thresholds in the SQ22536 plus EA-treated group were significantly less than those in the 10% DMSO plus EA-treated group from the 19th to 27th day after KOA induction (*P* < 0.001; Figure 4). However, there was no significant difference in the mechanical thresholds between the SQ22536 plus EA-treated group and the KOA group after SQ22536 injection (from the 19th to 27th day after KOA induction) (*P* > 0.05; Figure 4).

### Intrathecal Application of PKA Inhibitor H89 Reversed the EA Effect on Mechanical Allodynia

**FIGURE 5:**
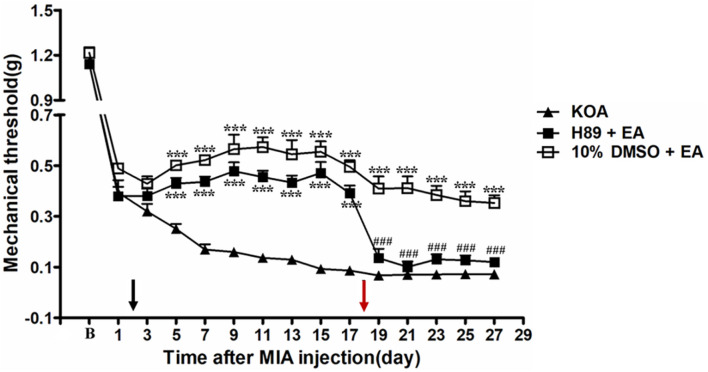
The effect of protein kinase A (PKA) inhibitor H89 on EA analgesia. Time course of the effect of PKA inhibitor H89 on tactile withdrawal threshold of EA mice. EA was administered for 30 min, once every other day for 4 weeks, starting from 2 days after MIA injection, as indicated by a black arrow. The PKA inhibitor H89 (2.5 μg) was administered i.t. 30 min before EA starting from 18 days after MIA injection, once every other day for five times, as indicated by a red arrow. Data are expressed as means ± SME (*n* = 8 mice in each group). ***P < 0.001, compared with the KOA group; ^###^*P* < 0.001, compared with the 10% DMSO + EA group.

The PKA inhibitor H89 (2.5 μg, i.t.) was administered 30 min before EA intervention starting from the 18th day after KOA induction, once every other day for five times. The H89 significantly reversed the EA effect on the tactile withdrawal thresholds (*P* < 0.001). As shown in Figure 5, the time course curves for these groups (KOA, H89 plus EA, and 10% DMSO plus EA-treated groups) significantly differed from those of the treatment group [*F*_(2,__315)_ = 393.87; *P* < 0.0001], across times [*F*_(14,__315)_ = 193.27; *P* < 0.0001], and for their interactions [*F*_(24,__315)_ = 8.08; *P* < 0.0001]. Further analyses indicated that the mechanical thresholds in the H89 plus EA-treated group were significantly less than those in the 10% DMSO plus EA-treated group from the 19th to 27th day after KOA induction (*P* < 0.01; Figure 5). Additionally, there was no significant difference in the mechanical thresholds between the SQ22536 plus EA-treated group and the KOA group after H89 injection (from the 19th to 27th day after KOA induction) (*P* > 0.05; Figure 5).

### Intrathecal Application of a Selective GABA_A_ Receptor Antagonist Bicuculline Reversed the EA Effect on Mechanical Allodynia

It has been reported that intrathecal injection of the GABA_A_ receptor antagonist bicuculline totally abolishes the anti-hyperalgesic effects of 5-HT_7_ receptor agonist in the CCI-SN model (Viguier et al., 2012). In order to explore whether GABA_A_ receptor is involved in the analgesic effect of EA, the GABA_A_ receptor antagonist bicuculline (0.1 μg, i.t.) was administered 30 min before EA intervention starting from the 18th day after KOA induction, once every other day for 5 days. We found that the GABA_A_ receptor antagonist bicuculline reversed the EA effect on the tactile withdrawal thresholds (*P* < 0.001).

**FIGURE 6:**
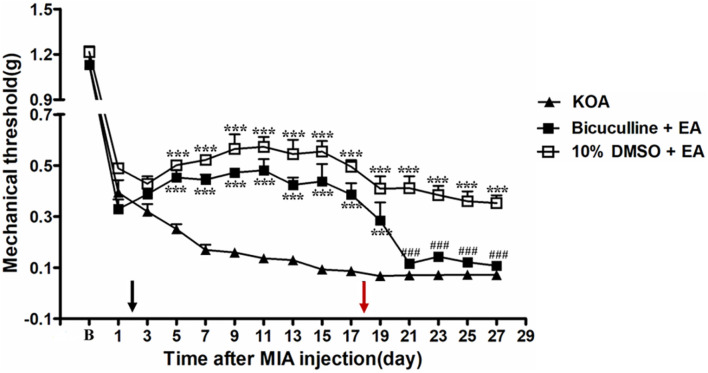
The effect of GABA_A_ receptor antagonist bicuculline on EA analgesia. Time course of the effect of GABA_A_ receptor antagonist bicuculline on tactile withdrawal threshold of EA mice. EA was administered for 30 min, once every other day for 4 weeks, starting from 2 days after MIA injection, as indicated by a black arrow. The GABA_A_ receptor antagonist bicuculline (0.1 μg) was administered i.t. 30 min before EA starting from 18 days after MIA injection, once every other day for five times, as indicated by a red arrow. Data are expressed as means ± SME (*n* = 8 mice in each group). ***P < 0.001, compared with the KOA group; ^###^*P* < 0.001, compared with the 10% DMSO + EA group.

As shown in Figure 6, the time course curves for these groups (KOA, bicuculline plus EA, and 10% DMSO plus EA-treated groups) differed significantly from treatments [*F*_(2,__315)_ = 335.60; *P* < 0.0001], across times [*F*_(14,__315)_ = 159.99; *P* < 0.0001], and for their interactions [*F*_(28,__315)_ = 6.74; *P* < 0.0001]. Further analyses revealed significant reduction in the mechanical thresholds in the bicuculline group when compared to those in the 10% DMSO plus EA-treated group from the 21st to 27th day after KOA induction (*P* < 0.001; Figure 6). Moreover, there was no significant difference in the mechanical thresholds between the bicuculline plus EA-treated group and the KOA group after bicuculline injection (from the 19th to 27th day after KOA induction) (*P* > 0.05; Figure 6).

## Discussion

A large body of evidence has demonstrated 5-HT_1_, 5-HT_2_, and 5-HT_3_ receptors to be involved in EA-mediated analgesia (Li et al., 2011; Seo et al., 2016). However, it is unclear whether the 5-HT_7_ receptor is also involved in this effect. In the present study, we found that KOA induction gradually reduced mechanical withdrawal threshold, and EA intervention significantly increased the tactile threshold after KOA modeling, which is consistent with our previous results (Yuan et al., 2018a, b). However, Sham EA had no significant effect on mechanical allodynia of KOA mice. Furthermore, results of western blotting revealed a significant increase in 5-HT_7_ receptor expression in the dorsal spinal cord after the administration of EA intervention. In the same vein, intrathecal administration of 5-HT_7_ receptor agonist AS-19 mimicked the analgesic effect of EA, while a selective 5-HT_7_ receptor antagonist reversed this effect. We have also proved that intrathecal injection of AC and PKA antagonists into the spinal dorsal horn prevented the anti-allodynia effect of EA. Similarly, intrathecal administration of GABA_A_ receptor antagonist bicuculline reversed the EA effect on pain hypersensitivity in the experimental mice. This study therefore provides clear evidence and suggests that the spinal 5-HT_7_ receptor may activate GABAergic neurons through the Gs–cAMP–PKA pathway to elicit pain inhibition and that the 5-HT_7_ receptor is involved in EA-mediated chronic pain inhibition in a mouse model of KOA.

The 5-HT receptor family is divided into seven subfamilies (5-HT_1_, 5-HT_2_, 5-HT_3_, 5-HT_4_, 5-HT_5_, 5-HT_6_, and 5-HT_7_), comprising 14 receptor subtypes, of which only 5-HT_1_, 5-HT_2_, and 5-HT_3_ receptors were shown to be involved in EA analgesia (Li et al., 2011; Seo et al., 2016). However, the role of other spinal 5-HT receptors in EA-mediated analgesia is yet to be elucidated. In this study, we have demonstrated that EA treatment reversed the reduced expression of 5-HT_7_ receptor in the dorsal spinal cord of KOA mice and proposed that spinal 5-HT_7_ receptor may be involved in EA-mediated analgesia.

Moreover, we have found that intrathecal injection of a selective 5-HT_7_ receptor antagonist SB-269970 30 min before EA intervention significantly reduced mechanical withdrawal thresholds, while SB-269970 injected alone into the spinal cord did not influence the pain threshold in our preliminary study, suggesting that the 5-HT_7_ receptor antagonist blocked the analgesic effect of EA in the KOA model. In addition, intrathecal injection of 5-HT_7_ receptor agonist AS-19 mimicked the analgesic effect of EA. Interestingly, this analgesic effect was more pronounced than that observed with EA treatment in the KOA model of chronic pain in mouse. This data corroborates the findings of Yang et al. (2014) who found that activation of 5-HT_7_ receptor by i.t. AS-19 exerted a significant antinociceptive effect on formalin-induced inflammatory pain models in rats. It is therefore reasonable to suggest EA pain inhibitory mechanism *via* activation of a descending serotonergic system that stimulates the release of spinal 5-HT, which in turn activates 5-HT_7_ receptor with subsequent inhibition of chronic pain.

It is well know that 5-HT_7_ receptor stimulates cAMP formation by activating AC *via* G, which also activates PKA and a series of downstream signaling pathways, leading to analgesic effect (Cortes-Altamirano et al., 2018). In the present study, we found that intrathecal administration of AC inhibitor SQ22536 30 min before EA intervention prevented the anti-allodynic effect of EA. Moreover, intrathecal injection of PKA inhibitor H89 30 min before EA reversed the EA effect on the tactile withdrawal thresholds. Our previous and preliminary studies found that AC inhibitor SQ22536 or PKA inhibitor H89 alone has any effect on the pain threshold in the central (Zhang et al., 2020) and spinal cord, which suggested that the AC–cAMP–PKA pathway may lack tonic activity, and the blocking effects of AC and PKA inhibitor on the EA-evoked inhibition were not a result of either compound facilitating nociceptive responses. These data further support that EA activates the spinal 5-HT_7_ receptor through the Gs–AC–cAMP–PKA pathway to inhibit chronic pain in a mouse model of KOA.

The co-localization of 5-HT_7_ receptor with spinal dorsal horn GABAergic neuron has been reported in a previous microscopic study (Lin et al., 2015). In support of this findings, an earlier study had reported that injection of GABA_A_ receptor antagonist bicuculline completely abolished the anti-hyperalgesic effect of 5-HT_7_ receptor agonist in the CCI-SN model (Viguier et al., 2012), which further suggests 5-HT_7_–GABAergic neuron coexistence. Our study has found that intrathecal injection of GABA_A_ receptor antagonist bicuculline prevented the anti-allodynic effect of EA, while bicuculline intrathecal injection alone did not influence the pain threshold in our preliminary study. Similarly, previous data have demonstrated that EA reduced neuropathic pain by increasing the protein level of GABA_A_ receptor in the dorsal horn of the spinal cord (Jiang et al., 2018). The present study therefore suggests that the spinal 5-HT_7_ receptor is involved in EA inhibition of chronic pain through activation of spinal GABAergic interneurons in a mouse model of KOA.

In conclusion, our study provided evidence to prove that the spinal 5-HT_7_ receptor is involved in EA inhibition of chronic pain *via* activation of GABAergic neurons through the Gs–cAMP–PKA pathway and therefore suggests 5-HT_7_-GABAergic neuron participation in EA-mediated inhibition of chronic pain in a mouse model of KOA. The present study also reported the 5-HT_7_–Gs–cAMP–PKA–GABAergic neuron pathway as a novel signaling pathway through which EA intervention leads to chronic pain inhibition in mouse model of KOA.

## Data Availability Statement

The raw data supporting the conclusions of this article will be made available by the authors, without undue reservation.

## Ethics Statement

The animal study was reviewed and approved by the Institutional Animal Care Committee of Xi’an Jiaotong University.

## Author Contributions

X-CY and F-QH conceived and designed the experiments. X-CY did most of the experiments and analyzed the data. X-JY and L-XT helped with the western blotting experiment. Y-LZ, Y-XG, HJ, and L-PX helped with mouse model and behavior test experiments. Y-YW, L-LL, LL, and HL helped with the data collection and analyzed the data. X-CY, SSB, and F-QH wrote the manuscript. All authors contributed to the article and approved the submitted version.

## Conflict of Interest

The authors declare that the research was conducted in the absence of any commercial or financial relationships that could be construed as a potential conflict of interest.

## Publisher’s Note

All claims expressed in this article are solely those of the authors and do not necessarily represent those of their affiliated organizations, or those of the publisher, the editors and the reviewers. Any product that may be evaluated in this article, or claim that may be made by its manufacturer, is not guaranteed or endorsed by the publisher.
